# The situated HKB model: how sensorimotor spatial coupling can alter oscillatory brain dynamics

**DOI:** 10.3389/fncom.2013.00117

**Published:** 2013-08-23

**Authors:** Miguel Aguilera, Manuel G. Bedia, Bruno A. Santos, Xabier E. Barandiaran

**Affiliations:** ^1^Department of Computer Science and Engineering Systems, University of ZaragozaZaragoza, Spain; ^2^Department of Informatics, CCNR, University of SussexBrighton, UK; ^3^Laboratory of Intelligent Systems, Cefet-MGBelo Horizonte, Brazil; ^4^Department of Philosophy, IAS-Research Centre for Life, Mind and Society, University School of Social Work, UPV/EHU University of the Basque CountrySan Sebastián, Spain

**Keywords:** HKB model, situated hkb model, embodied cognition, sensorimotor coupling, coordination dynamics, dynamical analysis, neurodynamics

## Abstract

Despite the increase of both dynamic and embodied/situated approaches in cognitive science, there is still little research on how coordination dynamics under a closed sensorimotor loop might induce qualitatively different patterns of neural oscillations compared to those found in isolated systems. We take as a departure point the Haken-Kelso-Bunz (HKB) model, a generic model for dynamic coordination between two oscillatory components, which has proven useful for a vast range of applications in cognitive science and whose dynamical properties are well understood. In order to explore the properties of this model under closed sensorimotor conditions we present what we call the *situated HKB* model: a robotic model that performs a gradient climbing task and whose “brain” is modeled by the HKB equation. We solve the differential equations that define the agent-environment coupling for increasing values of the agent's sensitivity (sensor gain), finding different behavioral strategies. These results are compared with two different models: a *decoupled HKB* with no sensory input and a *passively-coupled HKB* that is also decoupled but receives a structured input generated by a situated agent. We can precisely quantify and qualitatively describe how the properties of the system, when studied in coupled conditions, radically change in a manner that cannot be deduced from the decoupled HKB models alone. We also present the notion of *neurodynamic signature* as the dynamic pattern that correlates with a specific behavior and we show how only a situated agent can display this signature compared to an agent that simply receives the exact same sensory input. To our knowledge, this is the first analytical solution of the HKB equation in a sensorimotor loop and qualitative and quantitative analytic comparison of spatially coupled vs. decoupled oscillatory controllers. Finally, we discuss the limitations and possible generalization of our model to contemporary neuroscience and philosophy of mind.

## 1. Dynamicism and situatedness in cognitive (neuro)science

Cognitive science (and cognitive neuroscience in particular) is witnessing an increasing success of dynamical systems models, often displacing computational and representational conceptions of cognitive functioning. This change is not new, it can be traced back to early cybernetics (Ashby, 1952; Walter, 1963; Powers, 1973), neuroscience (Von Holst, 1973) phenomenology (Merleau-Ponty, 1942), and pragmatism (Dewey, 1896, 1922). But it was not until the 1990's that a strong paradigmatic shift began to take place in the fields of autonomous robotics (Brooks, 1991), adaptive behavior (Beer, 1990, 1997), coordination dynamics (Kelso, 1995), neuroscience (Skarda and Freeman, 1987), developmental psychology (Thelen and Smith, 1994), and philosophy of mind (Port and Gelder, 1995; Clark, 1997). Dynamicist approaches have had two central contributions: (a) that cognitive mechanisms (neural or otherwise) could be better effectively modeled and understood in terms of dynamical systems (Haken, 1978; Kelso, 1995; Freeman, 2001) instead of symbolic representational algorithms (e.g., Fodor, 1983; Pinker, 1997; Carruthers, 2006) and (b) that cognitive behavior could emerge out of recurrent sensorimotor loops in a self-organized manner, without the need for explicit encoding and planning on the side of the agent. And yet the relationship between both contributions remains relatively under-explored: how does the self-organization of behavior change the dynamical properties of brains? What is lost when we study brain dynamics in isolation from the sensorimotor loops they are naturally embedded in?

Some of the latest progress at both mechanistic (neurodynamic) and behavioral levels of dynamic modeling is related to *oscillatory dynamics*. Interactions between oscillatory components (neurons, brain regions, limbs or humans interacting with each other) are studied in terms of synchronization and phase-difference at various scales where macroscopic variables provide indexes of emergent collective behavior (Strogatz, 2004; Buzsaki, 2006). Oscillations are ubiquitous in nature, from planetary motion to circadian rhythms (Pittendrigh, 1960), from predator-prey populations (Lotka, 1920) to chemical dynamics (Kuramoto, 1984). Oscillatory activity is also present at different levels of the nervous system (Freeman, 2001). At the individual neural level, neurons undergo cyclic alterations on their membrane potential following different dynamical regimes depending on the cell properties (Izhikevich, 2006). At higher levels, global oscillations are observed as a collective phenomenon generated by groups of neural cells that fire synchronously [entrained by pacemaker cells or as a result of recurrent network activity with inhibitory-excitatory connections (Buzsaki, 2006)]. Different aspects of large-scale brain oscillatory activity (e.g., self-organization of emergent patterns, synchronization and oscillatory rhythms) have become a common explanatory resource in behavioral and cognitive neuroscience. Some of the phenomena that have gained explanatory benefit from this approach include the binding of the different perceived features of an object (Phillips and Singer, 1997), the representation of position information in navigation tasks (O'Keefe and Recce, 1993), attention (Deco and Thiele, 2009), memory (Jensen et al., 2007), and conscious experience (Crick and Koch, 1990; Engel et al., 1999; Varela et al., 2001).

Despite the significant progress recently achieved by investigating oscillatory dynamics in cognitive neuroscience, existing theoretical frameworks and models are mostly developed without taking into account sensorimotor dynamics and, even appear limited in the establishment of oscillatory correlations after a given stimulus onset. Computational models are generally built without considering the body and the environment and often assuming a representational theory of brain function (that is, they assume that the main job of the brain is to create a representation or model of the environment, and focus on neuronal mechanisms capable of supporting the processing of such a model). As a result, the focus of oscillatory brain dynamics is often centered on those aspects of oscillatory activity that might carry information within the brain, without considering the coupled brain-body-environment dynamics. This is even true for non-representational approaches to cognition that acknowledge the theoretical relevance of situated cognition but conduct most of their studies in search for cognitive correlates in oscillatory brain activity leaving aside the potential effects of the sensorimotor coupling (e.g., Skarda and Freeman, 1987; Varela et al., 2001).

Sensorimotor coordination implies more than the statement that sensory input will, through its influence on brain oscillations, create an action that, in turn, will produce a change that leads to a new perceptual state. The central claim of situated approaches to cognitive behavior is that the agent-environment coupling shapes brain dynamics in a manner that is essential to behavioral or cognitive functionality (Steels, 1990; Chiel and Beer, 1997; Clark, 1997). In other words, macroscopic functional behavior (e.g., intentional grasping or perception) emerges from microscopic sensorimotor dynamics (e.g., proprioceptive and visual feedback in grasping or saccadic movements in visual perception). Thus, cognitive behavior is not the result of a linear computational sequence involving sensation→perceptive-categorization→planning→ action-selection→motor-execution, but the result of recurrent sensorimotor and brain oscillatory coordination at multiple scales. The central role that sensorimotor dynamics play in cognitive phenomenology has been recently highlighted by Sensorimotor Contingency Theory (O'Regan and Noë, 2001), defending that what is constitutive of perceptual awareness (and, it could be argued, other cognitive states) is not a specific internal state of an agent, but the structure of sensorimotor contingencies. To perceive is to act in a specific manner that brings forth the structure of sensory changes in relation to the activity of the agent. To see or to perceive is something that is done and lies on the very sensorimotor coupled dynamics of an agent.

Filling the gap between the study of brain oscillatory activity and the situated sensorimotor dynamics is essential if we want to understand the nature of cognition. However, despite the repeated emphasis on the importance of sensorimotor coupling for neurodynamic approaches^1^ (Kelso, 1995; Freeman, 2001; Dreyfus, 2007; Chemero, 2009), there are very few examples of these types of models that exploit sensorimotor coupling and almost none for oscillatory models (some recent exceptions include Moioli et al., 2010; Santos et al., 2011). Current understanding of brain oscillatory dynamics is limited to “passive” conditions. The dynamical properties of oscillatory networks (even when studied within the context of behavioral or cognitive neuroscience, see Strogatz, 2004) are deduced from mathematical and computational models that have constant or no input at all, and the effect of sensorimotor or situated dynamics on the oscillatory properties of such networks is rarely considered. The goal of this paper is to make a theoretical contribution in the direction of explicitly quantifying the difference between dynamics that result from isolated vs. situated oscillatory controllers, and those that result from actively vs. passively coupled systems.

We have chosen the Haken-Kelso-Bunz (HKB) model as a paradigmatic example of oscillatory dynamics and behavior to address these questions. There are a number of good reasons to choose the HKB model. On the one hand the HKB model is simple enough to be treated analytically, on the other hand it has been used both to model behavioral phenomena and to model brain dynamics (see next section for details). Finally, to our knowledge, no variation of the HKB model exists that has used it as a controller of a sensorimotor system and no analytic study exists of a comparison between the dynamics of the HKB studied in isolation (with a parametric analysis) and its dynamics under sensorimotor loop conditions (with few exceptions as, for instance, Kelso et al., 2009).

The structure of the paper is as follows: (1) first we introduce the well known HKB model and the coordination dynamics paradigm; (2) next, we characterize the notion of dynamically coupled and spatially situated system and present a novel extension of the HKB model with sensorimotor embodiment that we call the *situated HKB* model; (3) then, we analytically solve a particular case of the situated HKB model performing a gradient climbing task in a 2D environment. Later, (4) we compare the obtained dynamics of the coupled system with the dynamics of a *decoupled HKB* and with a *passively-coupled HKB* model for an equivalent parametric analysis. Qualitative changes between the eigenvalues describing the three HKB-system dynamics will be identified, as well as experimental measures characterizing the transformation of the complete phase space of the agent produced by sensorimotor coupling. Finally, (5) we discuss some implications for the study of oscillatory brain dynamics.

## 2. The HKB model and the “coordination dynamics” paradigm

One of the most important current conceptual and modeling frameworks that might integrate oscillatory dynamics and sensorimotor coupling is Scott Kelso's *coordination dynamics* paradigm (Kelso, 1995) and the different variations of the *HKB model* (Haken et al., 1985) that have been used to study coordination phenomena. Coordination dynamics is a mathematical and conceptual framework used to investigate coordinated patterns in brain dynamics and behavior. It was proposed and developed by Kelso (1995), and is based on Haken's work on synergetics (Haken, 1978). It combines experiments and formal theoretical models to study how the components of a system interact and produce coherent coordination patterns.

The HKB model has been the driving example for the coordination dynamics paradigm, describing the behavior of two non-linearly coupled oscillators. The model was originally formulated in 1985 to explain experimental observations in the relative phase dynamics of bimanual coordination (Haken et al., 1985) but it has been shown to capture the coordination dynamics of different behavioral (Kelso, 1995), neural (Jirsa et al., 1994), and social (Oullier and Kelso, 2009) phenomena as well. Using the language of synergetics (order parameters, control parameters, instability, etc., see Haken, 1978), the HKB describes a simple non-linearly coupled dynamical system that captures the self-organized behavior of two generic coordinated nodes or units (Fuchs et al., 1995). More specifically, the HKB model was conceived to provide insights about: (1) the formation of ordered states of coordination; (2) the multistability of these states; and (3) the conditions that give rise to switching among coordinative states (Kelso, 1995). Moreover, the HKB model has been proven to describe fundamental features of self-organization such as multistability, phase transitions and hysteresis (Kelso, 1995).

In this paper we will use the “extended HKB” equation (Kelso et al., 1990),^2^ in which a system composed of two coupled oscillators is reduced to a single equation where the main variable is the relative phase between the two oscillators, and whose dynamics are shaped by the difference between the natural frequency of the oscillators and their coupling strength:

(1) $$\dot{\varphi }=\Delta \omega -a\cdot \text{sin}(\varphi )-2b\cdot \text{sin}(2\varphi )$$

**Figure 1:**
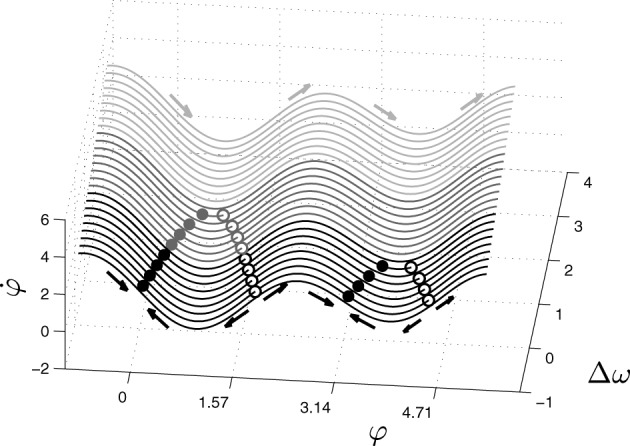
**Phase space of the extended HKB equation for fixed values of the coupling coefficients *a* and *b***. The system exhibits three different kinds of phase space depending on the control parameter Δω, showing multistable (black), monostable (dark gray) or metastable (light gray) dynamics. The filled and empty dots represent respectively the attractors and repellers of the system for different values of Δω. In this paper, the parameters used are choosen to ensure that the monostable region is the only one that is stable for the agent.

The relative phase or phase difference, φ, represents the order parameter or collective variable that emerges from lower-level interactions of the two coupled oscillators, *a* and *b* are the coupling coefficients between the two oscillators, and Δω is the difference between their intrinsic frequencies. Despite its simplicity, this equation captures a wide range of self-organized phenomena. Different combinations of the control parameters *a*, *b* (or rather *b*/*a*) and Δω give rise to different collective behaviors. For example, when shifting the value of Δω while the values of *a* and *b* are held fixed, the system experiences phase transitions between three different modes of behavior: monostable, bistable and metastable (Figure 1).

The HKB has been used to model different kinds of coordination phenomena but rarely used as a controller of an embodied agent. To be fair to the HKB model, that was never the intent of the original authors. The HKB model (and its extended version) was rather conceived to describe the behavioral dynamics at the macroscopic level (i.e., φ representing the collective variable of phase difference between two “behaving” oscillatory components, like fingers, oscillating armchairs in social coordination, etc.). It can be said that the HKB model was meant to capture the global agent-environment dynamics, not any explicit behavior generating mechanism that is coupled through sensors and motors to an environment. In addition, the HKB has also been used to model inter-areal coordination in the cortex (Tognoli and Kelso, 2009), ignoring the potential influence of the coupling between brain and environment. In sum, previous uses of the HKB model involve either full behavioral phenomena or “isolated” brain dynamics. However, there is a theoretical modeling gap that remains under-explored: the HKB as a controller of an agent that could modulate the control parameter (influenced by sensory input) through the behavior it generates when embodied in a robot. By filling in this gap we can address the following questions: How does the HKB model change its properties when situated (i.e., under closed-loop sensorimotor coupling in a spacial environment)? Is there any qualitative change that comes out of this coupling? Can the behavioral properties of an oscillatory “brain”, or controller, be deduced from the study of the brain in isolation or under constant input? Or even from variation of the input corresponding to those found in the coupled system? In the next section, we will try to provide answers to these questions by modeling a “situated HKB” and analytically solving the coupled agent-environment system and comparing it with isolated and passively coupled conditions.

## 3. Situated HKB model

### 3.1. Sensorimotor embodiment of the HKB equation

**Figure 2:**
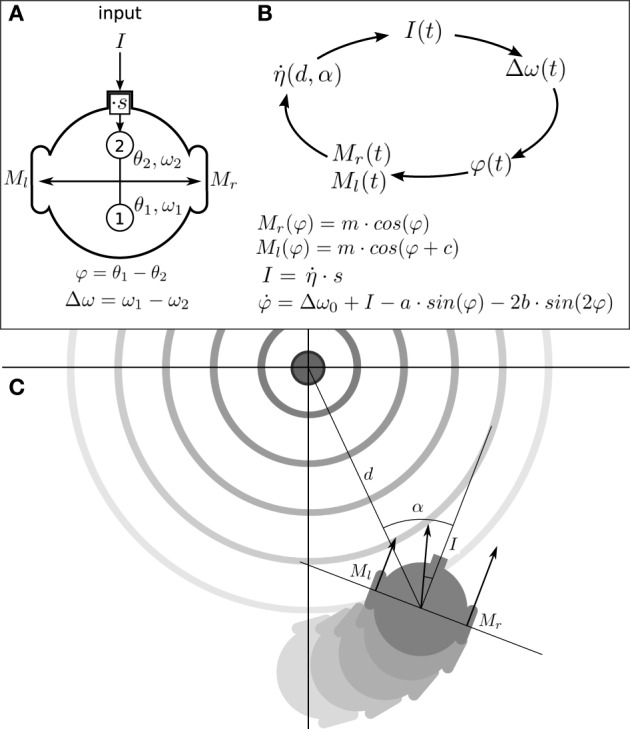
**Situated HKB agent. (A)** Structure of the agent, consisting in a sensor, two oscillatory controllers, and two motors. **(B)** Sensorimotor loop of the agent. **(C)** Representation of the agent interacting with its environment. The position and orientation of the agent respect to the center of the gradient are represented through the variables *d* and α.

In this section we describe what we have called the situated HKB system: a robotic model where the HKB equation describes the “neural system” of the agent which is embodied with sensors and motors and, in turn, situated in an environment. The agent has circular body of radius *R* with two diametrically opposed motors (see Figure 2A), that can move forward or backwards with different velocities in a 2D arena, and it has only one sensor that provides an input to the HKB neural controller.

Thereby, the HKB equation provides the macroscopic description of the dynamics of the two coupled oscillators. It allows us to describe the behavior of the situated HKB system in the following manner: (1) the agent has a “brain” where two regions (e.g., sensory and motor cortex) oscillate with their corresponding natural or intrinsic frequency (the difference between these frequencies is expressed by Δω_0_); (2) when a sensory input *I* modifies the natural frequency of one of the oscillators (e.g., sensory cortex), the frequency difference term changes;^3^ (3) since the frequency difference term is the control parameter of the phase difference, φ, we can consider that the situated HKB agent modulates its control parameter through sensorimotor contingencies: i.e., through the sensory changes that result from motor actions and the displacements they generate.

The dynamics of our agent is driven by:

(2) $$\dot{\varphi }=(\Delta \omega_{0}+I)-a\cdot \text{sin}(\varphi )-2b\cdot \text{sin}(2\varphi )$$

It is assumed that the agent is situated in a two-dimensional environment where a radial gradient of a stimulus η is present with its peak on the origin of coordinates (one can interpret this environment in different manners, e.g., as a light source or a chemical gradient that diffuses from its center symmetrically in all directions).

With regard to the “sensory system” of the model, since the agent lives in a world of gradients, we designed its sensor not to perceive the absolute amount of stimulus present in the environment but its change. Thus, the agent is sensitive to changes in η mediated by a sensitivity factor *s* that characterizes the sensor gain:

$$I=\dot{\eta }\cdot s$$

With respect to the “motor system” of the model, we define the activations of the motors as functions of the state of the controller,

$$\begin{array}{l} M_{r}(\varphi )=m\cdot \text{cos}(\varphi )\\ M_{l}(\varphi )=m\cdot \text{cos}(\varphi +c) \end{array}$$

where *M*_*r*_ and *M*_*l*_ represent the right and left motors, respectively, *m* is a speed parameter, and *c* is a bias parameter that breaks the symmetry between the right and left motors.

In this way, the brain-body-environment coupling can be understood as a process that repeats a cycled-course of four stages involving successive transformations of *I*(*t*), Δω(*t*), φ(*t*), *M*(φ(*t*)) and back to *I*(*t*) (see Figure 2B). To fully describe the movement of the agent (see Appendix 6 for a complete derivation of the agent's description) we include an additional variable describing the angle α of the agent's orientation relative to the peak of the gradient (see Figure 2C).

In Appendix A, the reader can find detailed information on the mathematical assumptions that we have considered for simplicity. As a direct result of assuming radial symmetry in the problem: (1) we can use a polar coordinates system (distance *d* of the agent to the center of the gradient, and angle α of orientation of the agent relative to the peak of the gradient) as the reference frame, and (2) it is considered that the variation of the gradient in terms of polar coordinates does not depend on the angle, $\dot{\eta }(d,\alpha )=\dot{\eta }(d)$.

The process can be characterized as follows : (1) with the movement of the agent in the environment (variation of *d*) the sensor receives a new input *I*, (2) the input influences the firing rate of the oscillators, changing the frequency difference between the HKB nodes (variation of Δω); (3) these frequency difference translates to a change in the phase difference between the oscillators (variation of φ) and, finally, (4) the new value of the phase difference changes the state of the motors [variation of *M*(φ)] moving the robot (variation of α) and starting the cycle again.

In terms of polar coordinates and substituting values (see Appendix A), the behavior of the agent {φ(*t*), *M*(φ(*t*))} can be represented by a reduced set of equations describing the system-environment coupling:

(3) $$\begin{array}{l} \dot{\varphi }=1+\dot{\eta }\cdot s-a\cdot \text{sin}(\varphi )-2b\cdot \text{sin}(2\varphi )\\ \dot{\eta }=\text{cos}(\alpha )\cdot (\text{cos}(\varphi )+\text{cos}(\varphi +c))\\ \dot{\alpha }=-\text{sin}(\alpha )/\eta \cdot (\text{cos}(\varphi )+\text{cos}(\varphi +c))+(\text{cos}(\varphi )-\text{cos}(\varphi +c)) \end{array}$$

where *a*, *b*, *c*, and *s* are the parameters of the system.

#### 3.1.1. Behaviorial analysis

We have chosen a basic a gradient climbing^4^ task for our agent to solve. That is, we ask the agent to climb up a linear gradient and maintain itself as close as possible to the maximum peak. A simple trial-and-error hand-tuning of the parameters gives us combinations that perform the desired behavior. We chose to adjust the parameters to *a* = 5, *b* = 1, and *c* = 5, leaving unspecified the sensitivity parameter *s* in order to have one free parameter to explore different kinds of behavior. This selection is arbitrary (except for the relation of *a*/*b*, which was chosen to ensure that the HKB is always in a monostable mode of functioning) but other combinations of parameters which result in gradient climbing behavior lead us to similar results in the analysis. For the experiments, the value of *s* will be defined in an interval of [0, 15].

**Figure 3:**
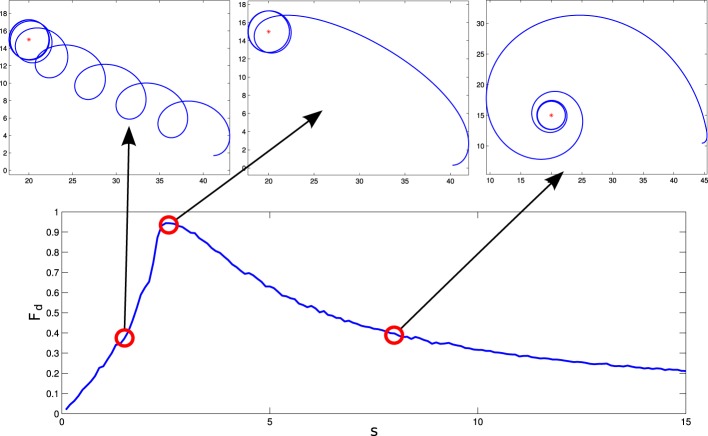
**Different behaviors performed by the situated HKB system**. We observe how different gradient climbing strategies arise depending on the value of s. For *s* = 1.5, the agent follows a cycloidal trajectory continuously turning over itself, for *s* = 2.5 the agent finds a direct path toward the peak of the gradient, and for *s* = 8 the agent slowly approaches the gradient peak following an spiral path.

For these parameters, we see that the agent displays different behavioral strategies depending on the value of its sensitivity parameter *s*, ranging from: (1) for values of *s* ∈ [0, 2.4] displaying cycloidal strategies where the agent turns over itself with a corkscrew-like movement, to (2) spiral paths where the agent slowly climbs the gradient, when *s* ∈ [2.6, 15]. At the frontier between these two behavioral strategies, we find (3) a critical region (*s* = 2.5) where the agent displays the most efficient gradient navigation (in terms of time and trajectory efficiency), taking a curved approximation path ending in an spiral-circular pattern around the peak of the gradient. These different behaviors are shown in Figure 3, where the efficiency of each gradient climbing strategy is computed with a parameter $F_{d}=1-\frac{d(t=t_{1})}{d(t=0)}$, that represents how close the agent gets to the center of the gradient in a given time (it is taken *t*_1_ = 40 s).

How do these behavioral strategies work? In the critical region (*s* = 2.5) the agent maps the highest gradient “sensation” with high activation of both motors, moving effectively toward the peak of the gradient. As we increase the value of *s*, sensory stimulation is more intense, so the agent needs an strategy where the approaching to the gradient peak is slower in order to maintain a compensated activation between the motors. On the other hand, when the value of *s* is decreased, the agent experiences difficulties to maintain an equilibrated velocity for both motors, having to turn around periodically to find again a trajectory where a high sensor input is perceived.

### 3.2. Analytical solution for the situated-HKB system

We shall now analytically solve the coupled brain-body-environment system in order to understand the emergence of the qualitatively different kinds of behavior that appear as we increase the sensitivity *s* of the agent. As usual, if we want to understand the behavior of an artifact modeled by a dynamical system, we will need to calculate the linearization of the system around its fixed points (Strogatz, 2001).

Thus, if we take the situated-HKB system of equations to be solved,

(4) $$\begin{array}{l} \dot{\varphi }=(1+\dot{\eta }(d,\alpha )\cdot s)-a\text{ sin}(\varphi )-2b\cdot \text{sin}(2\varphi ))\\ \dot{\eta }=\text{cos}(\alpha )\cdot (\text{cos}(\varphi )+\text{cos}(\varphi +c))\\ \dot{\alpha }=-\text{sin}(\alpha )/\eta \cdot (\text{cos}(\varphi )+\text{cos}(\varphi +c))+(\text{cos}(\varphi )-\text{cos}(\varphi +c)) \end{array}$$

where the parameter *s* (sensitivity) will be used as a control parameter to analyse the solutions in our range of interest *s*∈ [0, 15], it is easy to find that two fixed points can be obtained: (1) the first one is an attractor with values of φ, η, α at (0.11, 2.28, −π/2) and (2) the second one is a repeller at (2.53, 0.43, π/2).

**Figure 4:**
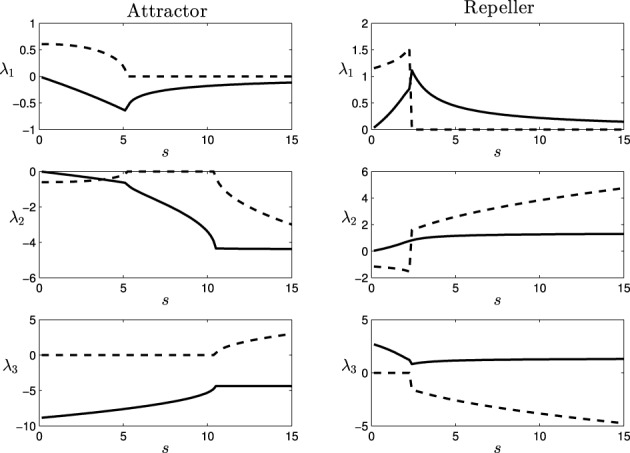
**Eigenvalues (λ_1_, λ_2_, and λ_3_) of the attractor (left) and repeller (right) fixed points of the situated-HKB: force of the attraction/repulsion vs. variation of the control parameter *s***. Real part (solid), Imaginary part (dashed).

Computing the Jacobian matrix of the system at the fixed points, and making an eigenvectors/eigenvalues analysis, we get the behavior of our dynamical system around the regions of its state space that bear qualitative significance. In Figure 4, it is illustrated the range of different values of the eigenvalues (denoted by λ_1_, λ_2_, and λ_3_) at each of the fixed points, depending on the parameter *s* (that corresponds to different observed behavioral patterns for gradient climbing, see Figure 3). We find regions that present simple attractor/repulsion dynamics (when λ_1_, λ_2_, λ_3_ are real numbers) whereas other regions present spiral attractions/repulsions (when λ_1_, λ_2_, λ_3_ have complex values).

**Figure 5:**
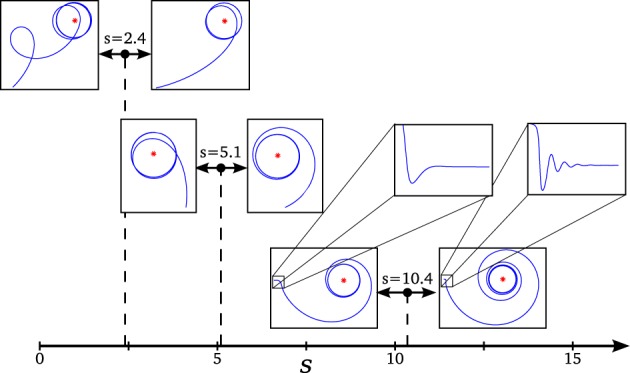
**Transitions in the phase space and changes in the agent's behavior**. We can observe how changes in behavior coincide with transition points in the dynamical description of the system: (*s* = 2.4) an abrupt transition of the repeller's plane of spiral repulsion explains the switch from cycloidal to stable movement in the agent, (*s* = 5.1) as the effect of spiral attraction vanishes, the agent changes from entering from inside to the final circular trajectory to entering from outside, and (*s* = 10.4) when the spiral attraction appears in a different plane, the agent displays an oscillatory movement for adjusting the followed trajectory (the effect of the oscillations is shown over the value of α in the enlarged box, its amplitude is damped so fast to be appreciated in the plot of the agent's trajectory).

In the following (see Figure 4), we show a detailed description of the relations between eigenvalues and behavior, analyzing the transitions of the eigenvalues in both the attractor and the repeller and focusing on the correspondences between those transitions and the respective transitions in the behavioral modes (Figure 5). Concretely, we analyse the transitions from real to complex eigenvalues (from regular attraction to spiral attraction), and behavioral transitions from underdamped to overdamped behavior (the system finds equilibrium with or without oscillating) on one hand and from spiral to cycloidal movement of the agent on the other.

#### 3.2.1. Attractor

- **Transition at** *s* = 5.1: At this point, the attractor experiences a change in its dynamics. An spiral attraction in the plane λ_1_λ_2_ disappears and the attraction of the system has no longer a spiral shape. At the same point, the approaching strategy of the agent experiences a change. For *s* < 5.1 the agent enters the final stable circular trajectory from within, whereas for *s* > 5.1 the agent enters from outside the circle. This approaching strategies correspond to a under-damped and a over-damped behavior of the {φ, η, α}−system, respectively.
- **Transition at** *s* = 10.4: The attractor changes again into a spiral shape, now in the plane λ_2_λ_3_. The behavioral change here is more subtle. It appears at the initial turning behavior of the agent until it finds an stable trajectory and enters into the spiral trajectory of the robot. When *s* < 10.4, the robot enters into the trajectory by softly adjusting the value of α with an over-damped behavior, while when *s* > 10.4 the value of α oscillates around the trajectory, adjusting to the optimal value with an under-damped behavior. This damping behavior also affects φ and η. Oscillations are too small to be clearly appreciated in the trajectory of the robot. That is why in Figure 5, in the enlarged boxes, we just represent the orientation of the agent α, which shows how the robot adjusts its behavior to the final trajectory.

#### 3.2.2. Repeller

- **Transition at** *s* = 2.4: At this point, a different kind of transition takes place. While transitions in the attractor were gradual, the change in the repeller at this value is an abrupt bifurcation. The system suddenly changes from a spiral in the plane λ_1_ λ_2_ to a spiral in the plane λ_2_ λ_3_. Also, a redistribution of the values of the real part of λ_1_ and λ_3_ (i.e., the “strength” of the repulsion) takes place in the transition. Consequently, the change in the agent's behavior is more dramatic in this case. As we saw in Figure 3, the agent changes from a cycloidal trajectory to a more stable strategy where the agent continuously approaches to the gradient source (either directly or following more pronounced spirals as *s* increases).

## 4. Comparison between situated, passively-coupled and decoupled HKB systems

In the previous section we have provided a full dynamical analysis and understood how, in the situated HKB, the coupled brain-body-environment system gives rise to a gradient climbing behavior. We also analyzed the transitions that take place as we increase the sensitivity parameter.

**Figure 6:**
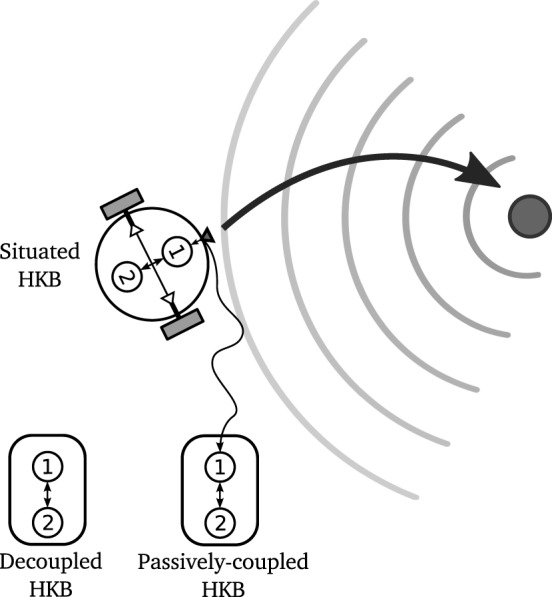
**Representation of a situated, decoupled and passively-coupled HKB systems**. The situated HKB system receives an input from its interaction with a gradient source. The passively-coupled HKB receives the copy of the input generated by a situated HKB. The decoupled HKB receives no input at all.

Now, we want to explore the effect of the sensorimotor situatedness of the system (i.e., the role of closed sensorimotor loop) upon the dynamics of the HKB equation by comparing the situated HKB with two homologous systems (see Figure 6):

1. A decoupled HKB system, as the one originally used by Kelso, in which the effect of situated interaction with an external environment is not taken into account.
2. A passively-coupled HKB system, where the HKB equation receives a structured input resulting from a real interaction between a situated HKB system and its environment, but where this input does not directly correspond to the activity of the system but is received from a recording or virtual input of a truly behaving agent^5^.

In the following subsections, we analyse the dynamics of these HKB models, under equivalent parametric conditions, searching for the qualitative difference that highlight the functional and neurodynamic significance of the closed sensorimotor loop.

### 4.1. Case 1: no sensory input—the decoupled HKB system

The decoupled HKB system simply consists in the classical HKB Equation (1) whose dynamics have been widely analysed. Given the classical HKB equation with the parameters used above for the situated HKB (Δω_0_ = 1, *a* = 5, and *b* = 1) and removing the sensory input, we get:

$$\dot{\varphi }=\Delta \omega_{0}-a\cdot \text{sin}(\varphi )-2b\cdot \text{sin}(2\varphi )$$

**Figure 7:**
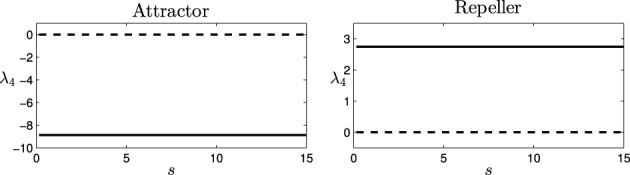
**Eigenvalues for the decoupled HKB (λ_4_): Force of the attraction/repulsion vs. variation of the control parameter *s***. Real part (solid), Imaginary part (dashed).

It is easy to see that, for this equation, two fixed points (or equilibrium points) are obtained by finding which values of φ make $\dot{\varphi }$ = 0. The fixed or equilibrium points are found at φ = 0.11 and φ = 2.53. Computing the Jacobian matrix of the equation for these values of φ, *J*(0.11) = −8.87 (attractor), and *J*(2.53) = 2.75 (repeller), provides us the values for the eigenvalue of the decoupled HKB at each point (denoted as λ_4_ and represented in Figure 7).

As we see, the eigenvalue is a real number at each fixed point, generating a simple pattern of attraction/repulsion in the dynamics of the system. Thus, the decoupled HKB alone cannot explain the behavioral changes shown in Figure 4, where simple patterns of attraction/repulsion were transformed into spiral cycles, or abrupt changes arise changing the plane of the resulting patterns. The decoupled HKB system displays simple attraction and repulsion forces around every fixed point and, therefore, the dynamics of the system are going to be described by constant attraction and repulsion forces regardless of the value of the parameter *s*.

In the situated HKB, we observed how the system displays “qualitatively different” behaviors, that is, behaviors that are not just due to gradual variations of a single dynamical regime but as a consequence of a phase transition in the system dynamics. This is a phenomenon that also appears for the original HKB equation (under some conditions the system is able to switch from one attractor to another, see Kelso, 1995). However, the situated-HKB with the parametric configuration used here (*a* = 5 and *b* = 1) φ remains within the monostable region (except for brief instants of time where the system visits the metastable region before stabilizing in the monostable). Thus, it cannot display the phase transitions observed in the bistable configuration of the HKB. Taking the decoupled HKB as a reference, the situated HKB should not present, in principle, qualitative changes that are not due to external factors.

Thus, the observed phase transition in the situated HKB system cannot be explained by the dynamics of the HKB model alone. Instead, the reason of this transition lies in the joint dynamics of the agent-environment system, as we illustrated when we solved the eigenvalues of the system. However, it is true that, in a certain sense, the difference of dimensionality of the two models is enough to substantially modify the dynamics of the system, independently of the fact that these extra dimensions correspond to the agent or the environment. The very fact that the situated HKB has three dimensions instead of one makes both systems are somewhat incommensurable.

The eigenvalues that determine the qualitative evolution of the system cannot be translated or mapped from the situated to the decoupled conditions: the whole brain-body-environment system defines a new eigenvalue coordinate system where the “brain” contribution cannot be isolated. The main issue is that we are talking about different systems: one consists of a single differential equation and the other of three coupled differential equations. It is hard if not impossible to compare the dynamics of a one dimensional system with the dynamics of a three dimensional system.

**Figure 8:**
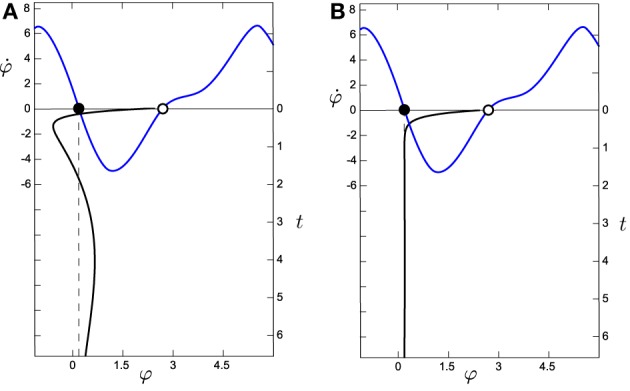
**Comparison of the evolution of the system around the attractor for (A) the situated HKB with ***s*** = 2.5 and (B) the decoupled HKB (with Δω = 1, both with ***a*** = 5 and ***b*** = 1)**. The black line in the vertical axis represents the evolution over time (right vertical axis) of φ, which has been simulated during 6.5 s with an Euler step of 0.1 with arbitrary initial values of φ = 0.65, η = −2.78, and α = −2.07. The blue line represents the phase space of the HKB, representing the attractors as filled dots and the repellers as empty dots. We observe how the decoupled HKB is only affected by a simple attraction force with constant strength, while a much richer dynamics is shown in the situated HKB, where different forces of attraction interact to modulate the systems evolution.

The decoupled HKB is affected by a constant force of attraction/repulsion (see Figure 8A) while the situated HKB is subject to forces in three different dimensions that continuously modulate each other (Figure 8B). Note that even if we were inducing a constant input (anywhere in the input range displayed by the situated system) the result will be equivalent. The next logical step is to question whether the crucial factor when comparing the HKB and the situated HKB systems is the specific structure of the input. In order to address this question we introduce the passively-coupled HKB model where the HKB equation receives the exact same input as the freely behaving situated HKB, but whose output has no effect.

### 4.2. Case 2: externally structured sensory input—the passively-coupled HKB system

We can model the passively-coupled HKB system by just adding a new variable φ^*^ that receives the same input as φ [i.e., a new equation ${\dot{\varphi }}^{*}=1+\dot{\eta }\cdot s-a\cdot \text{sin}(\varphi^{*})-2b\cdot \text{sin}(2\varphi^{*})$ is held together with the previous system (see Equation 4)]. As a result, we have to solve a four-dimensional system under the same conditions than before (parameters *a* = 5, *b* = 1, and *c* = 5, and the value of *s* defined in [0, 15]).

Analogously to the previous system we get two fixed points, an attractor at (0.11, 2.28, −π/2, 0.11) and a repeller at (2.53, 0.43, π/2, 2.53), and through the diagonalization of the respective jacobian matrices, four eigenvalues (λ_1_, λ_2_, λ_3_, λ_4_) are obtained.

As we will see below, although the term λ_4_ is decoupled from the activity of the situated HKB (and therefore independent of the type of coupling, i.e., independent of the value of s), the behavior of the new variable φ^*^ will necessarily be described by a combination of the eigenvalues of the situated HKB system (λ_1_, λ_2_, λ_3_) and the decoupled HKB equation (λ_4_). This will provide us a qualitative difference between the activity in φ and φ^*^.

In order to show it, we need to remind how the general solution of a dynamical system is built. It is well-known that the eigenvalues of a *linearized* dynamical system define the behavior of the system around the fixed points as a series of exponential functions which converge to an attractor or diverge from a repeller (Strogatz, 2001). For both the attractor and the repeller, the solution of any variable of the passively-coupled HKB system will have the form:

$$\begin{array}{l} x(t)=x_{0}+A_{\lambda_{1}}\cdot v_{1x}{\text{e}}^{-\lambda_{1}t}+A_{\lambda_{2}}\cdot v_{2x}e^{-\lambda_{2}t}\\ \;+A_{\lambda_{3}}\cdot v_{3x}e^{-\lambda_{3}t}+A_{\lambda_{4}}\cdot v_{4x}e^{-\lambda_{4}t} \end{array}$$

where *x* can stand for (φ, η, α or φ^*^), λ_*i*_ and *v*_*ix*_ in general have complex values (where *v*_*ix*_ are the eigenvectors of the solution and λ_*i*_ are the eigenvalues, *i* = 1, 2, 3, 4), *x*_0_ represents the position of the fixed point and *A*_λ_*i*__ are the coefficients that fix the initial state of the system (with *i* = 1, 2, 3, 4).

After making some simple calculations, we find that: (1) some eigenvectors are null (*v*_4φ_, v_4η_, v_4α_ = 0) so λ_4_ will only be part of the solution of φ^*^; (2) moreover, other ones share the same values (*v*_1φ_ = *v*_1φ^*^_, v_2φ_ = *v*_2φ^*^_, and *v*_3φ_ = *v*_3φ^*^_), so λ_1_, λ_2_, and λ_3_ will be equally present in the dynamics of φ and φ^*^, (3) finally, we can simplify the system a little more because (*v*_4φ^*^_ = 1) and (φ_0_ = φ^*^_0_) for any value of *s*.

Given that, the solutions of the system around *x*_0_ are simplified to:

$$\begin{array}{l} \varphi (t)=\varphi_{0}+A_{\lambda_{1}}\cdot v_{1\varphi }{\text{e}}^{-\lambda_{1}t}+A_{\lambda_{2}}\cdot v_{2\varphi }{\text{e}}^{-\lambda_{2}t}+A_{\lambda_{3}}\cdot v_{3\varphi }{\text{e}}^{-\lambda_{3}t}\\ \eta (t)=\eta_{0}+A_{\lambda_{1}}\cdot v_{1\eta }{\text{e}}^{-\lambda_{1}t}+A_{\lambda_{2}}\cdot v_{2\eta }{\text{e}}^{-\lambda_{2}t}+A_{\lambda_{3}}\cdot v_{3\eta }{\text{e}}^{-\lambda_{3}t}\\ \alpha (t)=\alpha_{0}+A_{\lambda_{1}}\cdot v_{1\alpha }{\text{e}}^{-\lambda_{1}t}+A_{\lambda_{2}}\cdot v_{2\alpha }{\text{e}}^{-\lambda_{2}t}+A_{\lambda_{3}}\cdot v_{3\alpha }{\text{e}}^{-\lambda_{3}t}\\ \varphi^{*}(t)=\varphi_{0}+A_{\lambda_{1}}\cdot v_{1\varphi }{\text{e}}^{-\lambda_{1}t}+A_{\lambda_{2}}\cdot v_{2\varphi }{\text{e}}^{-\lambda_{2}t}+A_{\lambda_{3}}\cdot v_{3\varphi }{\text{e}}^{-\lambda_{3}t}+\;A_{\lambda_{4}}\cdot {\text{e}}^{-\lambda_{4}t} \end{array}$$

Here, we can see that the dynamics of φ^*^ corresponds to the dynamics of φ plus an extra term which determines the difference between the situated and the decoupled model:

$$\varphi^{*}(t)=\varphi (t)+A_{\lambda_{4}}\cdot {\text{e}}^{-\lambda_{4}t}$$

This term, in the following, will be denoted as Δφ^*^(*t*)= *A*_λ_4__ · e^−λ_4_*t*^, representing the difference between the situated and passively-coupled HKB. We now quantify the influence of this extra term. Computing the solution in *t* = 0, we obtain that around the fixed points:

$$A_{\lambda_{4}}=\varphi^{*}(0)-\varphi (0)$$

That is, the influence of the decoupled term around the fixed points depends on the linear difference between the initial conditions of φ^*^ and φ.

Therefore, we can interpret the dynamics of the partially-coupled HKB, φ^*^(*t*), as composed of two “partially-decoupled” terms φ(*t*) and Δφ^*^(*t*) (partially decoupled because φ(*t*) influences Δφ^*^(*t*) but not the other way around). What is the difference between these two terms?

On the one hand, φ(*t*) follows a complex dynamic unfolding, intertwined with and modulated by the dynamics of η(*t*) and α(*t*) as a combination of the eigenvalues λ_1_, λ_2_, and λ_3_ (Figure 8A). On the other hand, the dynamics of Δφ^*^(*t*) are much simpler, defined by a unique eigenvalue λ_4_ (Figure 8B). However, we are analysing a highly idealized situation, where the system easily converges into its attractor without having to deal with any kind of perturbation.

In the subsection below we quantify the contribution of the Δφ^*^(*t*) term to the dynamics of the system in a more realistic situation. We analytically derive a theoretical expression to calculate Δφ^*^(*t*) in the presence of persistent perturbations and we validate the analytic results with numerical experimentation.

#### 4.2.1. Comparing situated and partially-decoupled HKB systems under perturbations

Typically, in a real system, variables are not subject just to different initial values as the term φ^*^(0) − φ(0) seems to represent. Variables in real systems are subject to continuous fluctuations in different forms. Each fluctuation in the difference between φ(*t*) and φ^*^(*t*) [that is, the difference between the fluctuations of φ(*t*) and the fluctuations of φ^*^(*t*)] is going to provoke an effect as function with the form *A*_λ_4__ · e^−λ_4_*t*^, with *A*_λ_4__ being equal to the amplitude in the fluctuation at time *t*, and λ_4_ the eigenvalue of the decoupled HKB in the attractor [thus assuming that the value of Δφ^*^(*t*) is small]. If fluctuations are present at different instants of time, the result will be a linear combination of all the exponential functions multiplied by the respective values of *A*_λ_4__ for each instant of time. If the fluctuations in the difference of φ(*t*) and φ^*^(*t*) are given by the function ξ(*t*), we can compute the final expression of the passively-coupled HKB around the fixed points as:

(5) $$\varphi^{*}(t)=\varphi (t)+\Delta \varphi^{*}(t)=\varphi (t)+\int_{0}^{t}\xi (\tau )\cdot {\text{e}}^{-\lambda_{4}(\tau -t)}\text{d}\tau$$

Without internal fluctuation the value of φ^*^(*t*) would converge to the value of φ(*t*) after an initial phase of adjustment. But if fluctuations are present, we can use the expression above to compare the fluctuations in the situated model with the fluctuations in the passively-coupled model.

**Figure 9:**
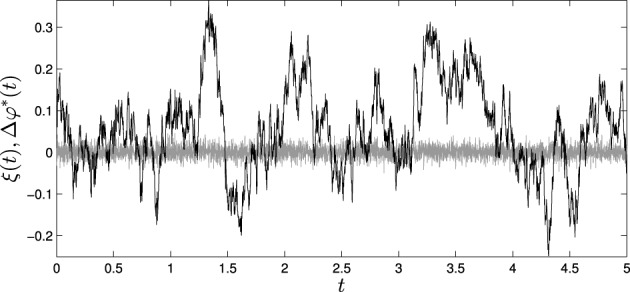
Effects of fluctuations in the passively coupled system: (gray line) ξ(*t*), difference of the introduced fluctuations in φ(*t*) and φ^*^(*t*) and (black line) Δφ^*^(*t*), fluctuations in the difference between φ(*t*) and φ^*^(*t*) computed through Equation (5).

As an example, we have simulated the situated HKB system with a passively-coupled HKB connected to it with an Euler step of 1 ms during a period of 5 s. We have introduced an additive white noise to the variables φ and φ^*^ with a variance of 10^−4^. Then, ξ(*t*) will be equal to the difference between these two sources of noise, which will conserve their white noise structure with twice its variance (2·10^−4^) (Figure 9, gray line). With Equation (5) we can compute the resulting fluctuation Δφ^*^(*t*) that will determine the differences between the values of φ(*t*) and φ^*^(*t*) (Figure 9, black line). We can observe how the fluctuations in Δφ^*^(*t*) have lost the uncorrelated white noise structure of the initial fluctuation, and now have a radically different structure with different temporal correlations induced by the e^−λ_4_*t*^ term. We can validate this result by comparing Δ φ^*^(*t*) computed with Equation (5) with the difference between φ(*t*) and φ^*^(*t*) measured experimentally without the effects reducing the system to a linear system around the attractor. That is, we can measure the error in the estimation of the fluctuation:

$$e(t)=(\varphi (t)-\varphi^{*}(t))-\Delta \varphi^{*}(t)$$

By computing *e*(*t*) we find that the amplitude of the error is significantly smaller than the amplitude of the theoretical measures of fluctuation Δφ^*^(*t*). Calculating the coefficient of determination for measuring how well the theoretical results adjust to experimental data, we obtain that (φ(*t*) − φ^*^(*t*)) fits Δφ^*^(*t*) with an *R*^2^ coefficient of 0.95, indicating a good fit of the data.

If we compute the variance of Δφ^*^(*t*) we find that it is equal to 12.3·10^−4^, that is, about 6 times bigger than the fluctuation introduced to the system. This tells us that if we are about to measure a passively-coupled version of the phenomenon we are interested in, we are to expect a bigger amount of fluctuation than in the real situated case. Furthermore, in the passively-coupled model fast fluctuations are modulated by slow fluctuations. And, whereas the original white noise introduced to the system can be averaged out and removed, the fluctuations added to the passively-coupled variable φ^*^(*t*) presents correlations at different scales that cannot be filtered easily.

#### 4.2.2. Dynamical signature

Above we have presented proof of the dynamical differences between a situated HKB system and a passively-coupled HKB system in the transients around the attractor and repellor of the system. As well, we have compared the results with experimental measures during the whole trajectory and without the effects of the linearization of the dynamical system, and demonstrated similar results. We have concluded that these changes are the product of the different modes of interaction of the situated and passively-coupled agent, which modulate the dynamical landscape of the brain-body-environment system. What do we mean when we say that the different types of coupling transform the dynamical landscape of the system? To clarify that we are going to analyse the *dynamical signature* of the HKB equation for the situated HKB and the passively-coupled HKB.

We can interpret this *dynamic signature* as the functional brain correlate of gradient climbing behavior. No single brain “state” (i.e., value of φ) is functionally significant in terms of behavior, what matters is the shape of the temporal pattern of phase relation among oscillators. In this sense, the “gradient-climbing behavior”, as a unit of explanation, is not the result of a set of brain states encoding a decision or a motor-program output, but results from a specific coordination pattern between sensor and motor surfaces, mediated by a specific coordination pattern between “brain oscillations”. The specific pattern of internal coordination that corresponds to gradient climbing behavior is here called its “dynamic signature”, the temporal structure of internal changes that is both cause and effect of different instances of a particular behavior.

To obtain and compare the dynamic signature of our agents, we have simulated the situated HKB system (with the sensitivity parameter *s* = 2.5) and a passively-coupled HKB connected to it, with an Euler step of 1 ms and a duration 10, 000 s, and we periodically reset the variables of the system (φ, η, and α) to new randomized values with intervals of 20 ms. The goal of this randomization is to sample a wide range of initial conditions of the system, that is, to capture a wide enough range of different situations that altogether constitute the abstract category of “gradient climbing behavior”. This way we can identify what a “neural signature” or dynamic pattern trace that corresponds to all the instances of this form of behavior. We formally define the dynamic signature of the HKB system as the density distribution of the derivative of the relative phase $\dot{\varphi }$ with respect to φ (or in terms of φ^*^ in the passively-coupled case).

**Figure 10:**
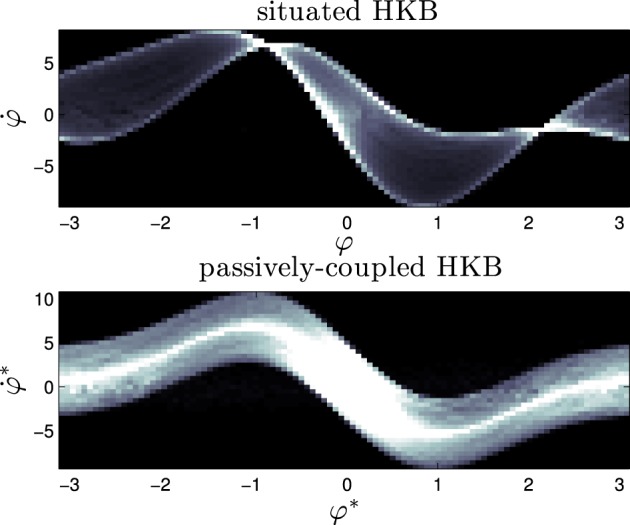
**Signature of the situated HKB with ***s*** = 2.5 and the corresponding passively-coupled HKB**. It represents the density distribution of the effective phase space of the HKB equation when it is coupled with an environment, showing the difference between situated and passive coupling.

What we see (Figure 10) is that the dynamical signature of the system changes severely when the system is situated in an environment. Whereas the passively-coupled HKB displays the shape of the original HKB phase space with a “blurring” effect created by the addition of an structured input (we can see it in Figure 10 as a “thick” line shaped with the form of the HKB original phase space), in the situated system the structure of the dynamical signature no longer resembles the original HKB phase space. The situated system has modulated or re-shaped its state space into a specific pattern through sensorimotor coordination.

## 5. Recapitulation, discussion, and interpretation of results

We have just shown how the HKB system displays qualitatively different dynamics under situated conditions, as compared to decoupled and passively-coupled conditions. We can recapitulate the main results as follows:

1. Transitions between qualitatively different types of sensorimotor behavior, that are generated by the situated HKB with increasing sensitivity, cannot be deduced from the behavior of the decoupled HKB nor from the analysis of the passively coupled HKB alone. The nature of these transitions can only be revealed through the analysis of the whole brain-body-environment system.
2. Even for a single fixed value of the sensitivity parameter the transient trajectory of the situated HKB system toward its attractor is far from trivial, it unfolds in different ways at different temporal scales. The transient trajectory of the decoupled HKB system, instead, is relatively simple and monotonic.
3. A passively-coupled agent receiving an input generated by a situated agent shows correlated and amplified fluctuations that are not present in the situated agent.
4. Finally, the dynamical signature of the agents shows us how the type of coupling (passive or situated) severely transforms the phase space of the HKB system. The specificity of functional neural signatures is lost when studying the brain out of the closed sensorimotor loop, even if it is subjected to exactly the same input.

These kinds of differences between situated and decoupled oscillatory controllers illustrate how much we would miss if we analysed the “brain” of an agent isolated from its embodied situatedness in an environment. On the one hand, the brain-body-environment system constitutes a dynamical holistic continuum where phase transitions can take place, without necessarily corresponding to phase transitions that would occur in an isolated brain. Quite the opposite, in general this joint dynamical structure would be hardly deduced from the isolated controller of an agent. On the other hand, the unfolding of behavior shown by a system is modulated by the continuous interplay with the environment at different time-scales that generally are not present in the dynamics of the controller system alone, giving rise to much richer behavioral dynamics.

Our situated HKB is a case of double coordination: sensorimotor coordination of the coordinated dynamics of the two oscillatory components (modeled by the HKB equation). What is crucial is the fact that *under situated conditions the dynamics of the HKB can be modulated by the precise and interactively structured coordination between its internal dynamics and the sensorimotor environment*. The mode in which sensory input (the control parameter) changes as a function of the motor output (which is in turn generated by internal dynamics) through the environment, makes possible this higher order coordination. It can be said that the agent modulates its internal dynamics through sensorimotor coordination, in a manner that is not available to the decoupled or passively-coupled system, resulting in functionally specific internal patterns that we have demonstrated with the dynamic signature.

We can learn about the HKB system as a model in (sensori)motor control and oscillatory brain dynamics. Notwithstanding are the important contributions that this model has brought with its application to behavioral and neural sciences. There are, however, important limitations on the type of modeling. The most relevant for us is that, for the paradigmatic cases (like finger coordination), there is no genuine sensorimotor coupling being modeled. A subject is asked to move the fingers in coordination with a metronome but this is the only coupling that exists. Finger movement is a motor task, driven by a sensory cue (the pace of the metronome) but it is not a sensorimotor task. It is unnatural to instruct or constrain habitual behavior in response to the tight instructions of an experimenter. Human and animal behavior is generally the result of a “free” sensorimotor coupling where every motor variation carries with it a sensory variation, bringing about a coordinated behavioral pattern. In terms of the finger coordination experimental paradigm, the “natural situation” would resemble one where a finger movement alters the metronome's pace, which in turn alters the finger movement, etc. The HKB model has been applied to other, more complex tasks (like social coordination) but, to our knowledge and despite the emphasis of many advocates of sensorimotor dynamics (Kelso, 1995; Chemero, 2001, 2009), there is no available model of HKB for sensorimotor coordination itself. It was therefore crucial to understand the dynamics of the situated HKB to explore in detail the way in which coordination dynamics can be radically altered when oscillatory dynamics appear coupled to sensorimotor dynamics. The HKB equation has been used to model cases of sensorimotor coupling such as (Kelso et al., 2009), where a human subject receives sensory feedback from a computer screen, and the human's behavior in turn affects the computer. The novelty of the situated HKB model is that the coupling is spatial and the HKB is not meant to capture the global feedback dynamics, but is used directly as a robotic controller. As well, whereas in the experiment above part of the interaction was an human subject, the situated HKB is a model which can be fully described by just three equations. This is a great advantage in terms of dynamical analysis, allowing us a much deeper analysis of the system's behavior.

It is important to note that the present paper has focused on the most simple configuration of the HKB model; with an extremely simplified body and environment. Regarding the internal configuration of the HKB, we have studied it under the simplest parametric configuration that produces a single attractor instead of two or none, which is due to the *a*/*b* coefficient value that we kept fixed. Even for this simple configuration we have found qualitative differences of the transient dynamics of the system before falling into the attractor—see φ = 0.11 and transient dynamics around (−1, 2.50) in Figure 8. The behavior of the system shows strong differences under the situated and decoupled conditions. But the HKB can display much richer behavior on its bistable configuration (with two attractors and repellers) or along the metastable region (see light gray area in Figure 1) where there are no fixed points but just attractor and repeller shadows (a phenomenon that appears for high values of difference between the natural frequencies Δω (Kelso, 1995) (something that did not occur on our model given the low value of Δω_0_).

The differences we found between decoupled, passively-coupled and situated dynamics can be expected to be amplified for richer parametric configurations of the HKB model. In fact, in a parallel paper (Santos et al., 2012), some of this paper's authors have studied the dynamics of the same situated HKB model performing a gradient climbing task under metastable regimes of φ. In this case the effect of sensorimotor modulation of the HKB dynamics was much stronger, shaping specific metastable regimes and transitions between them, whereas passively-coupled agents showed different regimes and transitions. We want to stress that the appearance of qualitative differences between situated and decoupled systems was not a contingent results of the parameter values chosen in this paper (nor those of Santos et al., 2012). We have observed the same changes for different parameter values and also with different oscillatory controllers (such as Kuramoto oscillators—unpublished results).

As the experimental setup is regarded, we have seen that a simple linear and radially symmetric 2D gradient environment, a single sensor and two motors were sufficient for the HKB to exploit the sensorimotor coupling so as to modulate its internal dynamics in qualitatively different manners under different coupling conditions. Richer environments, more complex tasks and, multimodality and higher dimensional sensory and motor surfaces could increase the divergences we have shown here. It has been shown that in cross-modal perception, perturbations to one sensory modality can be compensated by other sensory modalities (Ernst and Banks, 2002), it is therefore to be expected that multi-modal sensorimotor engagement could have an even greater effect on brain dynamics than a single sensorimotor modality.

Could these results be generalized to neuroscience? Not directly, we have just provided a proof of concept of how severely can oscillatory brain dynamics be altered by sensorimotor coordination. However, even if true for our extremely simplified model this conclusion has still to be proven for neuro-biological systems. We are not aware of any neuroscientific study comparing situated and passively coupled recordings for perceptuomotor tasks, but new recording techniques (Linderman et al., 2006; Santhanam et al., 2007; Fan et al., 2011) might help replicate the experiments we have developed in this paper. The cognitive or psychological effects of different degrees of disruption of the sensorimotor loop could range from a complete lack of perceptual capacity (e.g., when inducing sensory streams resulting from input recorded from saccadic exploration) to a loose sense of reality when sensorimotor coupling conditions in virtual reality are not optimal. New experimental paradigms in substitutional reality (SR) (playing back recorded visual experience to re-create realistic scenes) (Suzuki et al., 2012) have shown that “a major factor influencing successful substitution in the SR system was consistent visuo-motor coupling throughout the experience” (Suzuki et al., 2012, p.6).

At a more abstract level of discussion, the present model makes a theoretical contribution to the ongoing debate around the *causal* vs. *constitutive* role of action in perception. Roughly speaking causal theories (Prinz, 2006; Adams and Aizawa, 2008) claim that movement can perfectly be a cause of the right sensory input that in turn causes perceptual states but it is not strictly necessary. Constitutive theories on the other hand (O'Regan and Noë, 2001; Noë, 2004) claim that movement itself is part of the perceptual process. Causal theories are generally internalist by asserting that what matters is the brain state (caused by the sensory input), whereas constitution theories tend to align with externalism (perception is a process that involves a distributed set of brain, body and environmental components, all of them constituting the same percept). Our model can be used to show how, even if favoring internalism, the neural signature that corresponds to a given cognitive episode can be qualitatively different from the neural signature obtained when the very same input (cause) is induced into a passively-coupled system and fine grained sensorimotor contingencies become strictly necessary or constitutive of functionally distinct neural signatures. Whether this holds also true for natural systems is open to experimentation but the conceptual discussion, which is often obscured by a lack of clear models, can benefit from the findings presented here.

## 6. Conclusion

Contemporary neuroscience often assumes that it is possible to deduce the behavioral properties of the brain by just studying its dynamics under “passive” input conditions (e.g., neural recordings in anesthetized animals) or building models that ignore sensorimotor dynamics (like large scale networks with noise-inputs or otherwise non-behaviorally controllable input). The brain-body-environment coupled dynamics are rarely considered as a unified dynamical system and there is still a limited understanding of the interplay between sensorimotor and neural dynamics.

In this paper we have illustrated what neuroscience might be missing when ignoring the role of sensorimotor coordination, particularly when drawing models of brain dynamics out of neural recordings in the absence of closed sensorimotor loops. We have presented a minimal model that shows the qualitative differences that can arise under situated and decoupled sensorimotor conditions. Our analysis was centered in the HKB model, which is a widely accepted minimal model of neural and behavioral coordination, and is simple enough to facilitate a deep formal analysis of its dynamical structure. The HKB model has been widely used to explore both coordination dynamics of sensorimotor self-organizing phenomena and coordination dynamics in brain activity. But none of the previous variations and developments of this model has integrated both aspects. To fill this gap we have formalized the *situated HKB* system, where a sensory input modulates the control parameter, and the main variable of the model produces motor variations that in turn result in sensory input change. A detailed mathematical analysis of the situated HKB has shown that there is features of the model which cannot not be deduced from the analysis of the isolated or passively-coupled systems (even if it receives the exact same sensory input). We have shown how some features, such as (a) the phase transition that takes place modifying a sensitivity parameter, (b) the attraction patterns, (c) the neurodynamic signatures, and (d) the modulatory capacity of the situated system. All of them need to be explained by a framework that takes into account the coupled dynamics of the brain-body-environment system. How far these results can be generalized to experimental neuroscience remains open to experimentation, the present contribution was a theoretical one aiming at making a formal characterization and a proof of concept of how sensorimotor dynamics can alter the oscillatory coordination properties of behavior-generating mechanisms.

### Conflict of interest statement

The authors declare that the research was conducted in the absence of any commercial or financial relationships that could be construed as a potential conflict of interest.

## Figures and Tables

**Figure A1:**
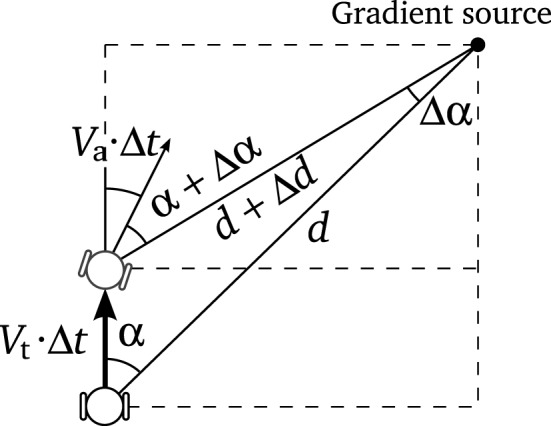
**The equations of the movement of the agent can be deduced from this schema**.

## Footnotes

^1^By neurodynamic approaches we refer to dynamical system approaches to the understanding of neural activity (Gelder, 1998; Freeman, 2001; Buzsaki, 2006, etc.).

^2^We shall hereafter use the terms “HKB model” or “HKB system” to refer to the “extended HKB model” (Kelso et al., 1990) rather than the original (and simpler) HKB model (Haken et al., 1985). The reason for this is that we are further going to distinguish situated, decoupled and passively-coupled versions of the extended HKB model and names would become far too long if referred to as, for example, “passively-coupled extended HKB”.

^3^The term Δω of the original extended HKB equation has been substituted by the term Δω_0_ + *I*, where *I* is the sensory input term whose effect is mathematically equivalent to changing the natural frequency of one of the oscillators (see Figure 2A).

^4^Gradient climbing is a minimal (yet not totally trivial) task, which is widespread in nature. Most of small scale adaptive behavior occurs along chemical gradients. The microscopic world is a world of gradients (like thermal gradients or light gradients but mostly chemical gradients). The adaptive behavior of small animals (e.g., *C. elegans*) and individual motile cells (e.g., bacteria but also animal cells migrating during development) is mostly a gradient-related adaptive behavior. Navigating smell or heat gradients is also a stereotypical adaptive task for higher animals. Moreover many instances of higher-level behavior can also be interpreted as abstract gradient climbing (e.g., a human can move up a gradient of social popularity or economic wealth that might involve complex strategic decisions combined with an emotional or sensitive gradient climbing of the perceived result of such strategies).

^5^A similar experimental setup in developmental neuroscience was carried out by Held and Hein (1963) where two kittens were reared by holding one immobile and attached to the other, so that both received the same sensory stimulation, yet only one had freedom to control movement.

## Appendix

### A.1. Development of the three-dimensional equation of the situated-HKB model

As we have seen, the behavior of the *situated* HKB agent will be completely addressed by the variable φ(*t*) and the position of the motors of the agent *M*(φ(*t*)) at each time *t*.

- **Assumption 1:** The choice of polar coordinates (*d* representing the distance to the source and α representing the relative angle between the agent’s orientation and the source), instead of cartesian coordinates, will allow us to simplify the dimensionality of the model.
  Assuming that the agent’s mass is small enough to be neglected (in order to avoid inertial resistance), we can describe the speed of the agent as follows: the translational speed of the robot *V*_*t*_ is calculated as the vectorial average of the motor velocities, and the angular speed *V*_*a*_ as the difference of the motor velocities divided by the body diameter).
  $$\begin{array}{l} V_{t}(\varphi )=(M_{r}(\varphi )+M_{l}(\varphi ))/2\\ V_{a}(\varphi )=(M_{r}(\varphi )-M_{l}(\varphi ))/2\cdot R \end{array}$$
  Knowing the tangential and angular velocities of the robot *V*_*t*_ and *V*_*a*_, we can deduce the movement of the agent in terms of polar coordinates (see Figure A1):
  Taking Δ*t*, Δ*d*, Δα → 0, we get
  $$V_{t}\cdot \Delta t\cdot \text{cos}(\alpha )+(d+\Delta d)\text{cos}(\Delta \alpha )=d$$
  from which, the next equation is easily obtained
  (6) $$\dot{d}=-\text{cos}(\alpha )\cdot V_{t}$$
  Similarly, if we take
  $$V_{t}\cdot \Delta t+(d+\Delta d)\cdot \text{cos}(\alpha +\Delta \alpha +V_{t}\cdot \Delta t)=d\cdot \text{cos}(\alpha )$$
  we find that
  (7) $$V_{t}+d\cdot \text{sin}(\alpha )\cdot (\dot{\alpha }+V_{a})+\dot{d}\cdot \text{cos}(\alpha )=0$$
  Putting together Equations (6) and (7), we obtain the movement equations:
  $$\begin{array}{l} \dot{d}=-\text{cos}(\alpha )\cdot V_{t}\\ \dot{\alpha }=\text{sin}(\alpha )/d\cdot V_{t}+V_{a} \end{array}$$
  where we know that *V*_*t*_ and *V*_*a*_ depend on the speed of the motors:
- **Assumption 2:** For simplicity, it is considered that η = *d*_0_ − *d*, where *d* is the distance to the center of the gradient and *d*_0_ is the intensity of the stimulus at the center of the gradient, which will decrease linearly with *d*.
  Since η and *d* are inversely proportional and radial symmetry allows us to dismiss the angle of the position of the agent respect to the gradient when we describe it in polar coordinates, so the change of the perceived gradient only depends on the position of the agent and the gradient itself,
  $$\dot{\eta }=\dot{F}(d,\alpha )=\partial F(d,\alpha )/\partial d\cdot \dot{d}+\partial F(d,\alpha )/\partial \alpha \cdot \dot{\alpha }$$
  and the set of equations describing the system-environment coupling can be reduce to only three:
  $$\begin{array}{l} \dot{\varphi }=\Delta \omega_{0}+\dot{\eta }\cdot s-a\text{ sin}(\varphi )-2b\cdot \text{sin}(2\varphi )\\ \dot{\eta }=\text{cos}(\alpha )\cdot (M_{r}(\varphi )+M_{l}(\varphi ))/2\\ \dot{\alpha }=\text{sin}(\alpha )/(d_{0}-\eta )\cdot (M_{r}(\varphi )+M_{l}(\varphi ))/2+(M_{r}(\varphi )-M_{l}(\varphi ))/(2R) \end{array}$$
- **Assumption 3:** Without loss of generality, we choose *m* = 2, Δω_0_ = 1, *d*_0_ = 0 and radius of the body *R* = 1, and we get:
  (8) $$\begin{array}{l} \dot{\varphi }=1+\dot{\eta }\cdot s-a\cdot \text{sin}(\varphi )-2b\cdot \text{sin}(2\varphi )\\ \dot{\eta }=\text{cos}(\alpha )\cdot (\text{cos}(\varphi )+\text{cos}(\varphi +c))\\ \dot{\alpha }=-\text{sin}(\alpha )/\eta \cdot (\text{cos}(\varphi )+\text{cos}(\varphi +c))+(\text{cos}(\varphi )-\text{cos}(\varphi +c)) \end{array}$$
  where *a*, *b*, *c*, and *s* are the parameters of the system.
